# Quality of Life in Patients With Methamphetamine Use Disorder: Relationship to Impulsivity and Drug Use Characteristics

**DOI:** 10.3389/fpsyt.2020.579302

**Published:** 2020-09-30

**Authors:** Yingying Wang, Jinsong Zuo, Wei Hao, Hongxian Shen, Xiaojie Zhang, Qijian Deng, Mengqi Liu, Zhiqiang Zhao, Lina Zhang, Yanan Zhou, Manyun Li, Tieqiao Liu, Xiangyang Zhang

**Affiliations:** ^1^National Clinical Research Center for Mental Disorders, and Department of Psychiatry, China National Technology Institute on Mental Disorders, Hunan Key Laboratory of Psychiatry and Mental Health, Institute of Mental Health and Hunan Medical Center for Mental Health, The Second Xiangya Hospital of Central South University, Changsha, China; ^2^College of Life Sciences and Chemistry, Hunan University of Technology, Zhuzhou, China; ^3^Department of Medicine Addiction, Xinjiang Mental Health Center and Urumqi Fourth People’s Hospital, Urumqi, China; ^4^Department of Psychiatry, Brains Hospital of Hunan Province, Changsha, China; ^5^CAS Key Laboratory of Mental Health, Institute of Psychology, Chinese Academy of Sciences, Beijing, China

**Keywords:** methamphetamine, quality of life, impulsivity, substance use disorder, drug abuse, drug use

## Abstract

**Background:**

The quality of life (QOL) of patients with methamphetamine use disorder (MAUD) is increasingly recognized as an important outcome. Previous studies have found that impulsivity is negatively associated with QOL in mental disorders, but this relationship is rarely confirmed in patients with MAUD. We hypothesized that impulsivity is negatively correlated with QOL in patients with MAUD based on previous findings. In addition, a variety of drug use characteristics of patients that may potentially affect their QOL need to be further explored. Therefore, the purpose of this study was to explore the relationship between impulsivity, multiple drug use characteristics, and QOL in patients with MAUD.

**Methods:**

A total of 379 patients with MAUD were recruited, and the majority of them were male (85.5%), with an average age of 33.93 ± 7.08 years. Two psychiatrists conducted semi-structured interviews with methamphetamine (MA) users in two compulsory drug rehabilitation centers to obtain their demographics and drug use characteristics. The Barratt Impulsiveness Scale-11 (BIS-11) and Brief WHO Quality of Life Assessment (WHOQOL-BREF) were used to assess patients’ impulsivity and QOL, respectively. Correlation and univariate regression analysis were used to explore the relationships between impulsivity, a series of drug use characteristics and patients’ QOL in different domains. Further multiple linear regression analysis was used to identify what extent the above clinical variables explained the variations in patients’ QOL.

**Results:**

Age, marital status, employment, and various drug use characteristics were significantly associated with at least one QOL domain. Among them, married and full-time job were positively correlated with QOL, while others were negatively correlated with QOL. The total score of BIS-11 was significantly negatively correlated with all four domains of QOL. Impulsivity, a range of drug use characteristics and certain demographic characteristics collectively explained varying degrees of variation in different domains of QOL.

**Conclusions:**

Impulsivity and various drug use characteristics can significantly predict QOL in all fields of MAUD patients. In addition, we have also found differences in the predictors of QOL in different domains. Overall, this study provides clinical guidance for the treatment of MAUD patients, that is, management of impulsivity in patients with MAUD may help improve their QOL and even sustain their drug rehabilitation.

## Introduction

Quality of life (QOL) refers to individuals’ subjective perception of their own life status, which involves the domains of physical health, psychological state, social relations and living conditions (1). In the past decade, QOL has been paid more and more attention by psychiatrists because of its importance for outcomes of patients with mental illness (2–4), especially for schizophrenia (5, 6), major depression (7, 8) and bipolar disorder (9, 10). In fact, QOL is not one of the traditional or formal measures of outcomes. Therefore, in contrast to these common psychiatric diseases, a more insidious condition named substance use disorders (SUDs), has received relatively little attention in terms of QOL from both clinicians and researchers (11). Despite the limited number of studies, it does not prevent evidence that QOL has increasingly become a determinant of the outcomes of patients with SUDs (12, 13). More specifically, it has been shown that improved QOL can effectively reduce the use of drugs during treatment seeking (14, 15). In addition, a high QOL contributes to the sustainable withdrawal of patients with SUDs after leaving treatment (16, 17). However, patients with SUDs often reported more impaired QOL than those without SUDs (18, 19), or even poorer than some other chronic disease (20, 21), which may greatly hinder their rehabilitation. Consequently, it is urgent for clinicians to understand the factors leading to the poor QOL in patients with SUDs, so as to improve it in a cost-effective manner.

Previous studies have consistently concluded that impulsivity was significantly associated with QOL, especially in a group of mental disorders characterized by impulsivity, such as bipolar disorder (9, 22), attention deficit hyperactivity disorder (23, 24) and emotional eating (25, 26). In fact, although impulsivity has been shown to be a non-core symptom, in popular diagnostic systems (such as DSM-5 and ICD-11), certain impulsive behaviors can still be used to diagnose SUDs, such as uncontrolled drug seeking (27, 28). Therefore, we hypothesized that the high impulsivity of patients with SUDs may have an impact on their QOL. Unfortunately, there are few studies on the relationship between impulsivity and QOL in patients with SUDs. Although rare, fortunately, we have drawn from this small number of studies that in the SUDs population, patients with higher impulsivity have worse QOL than those with lower impulsivity (29, 30). Furthermore, impulsivity may be a predictor of QOL in patients with SUDs. More specifically, high impulsivity is associated with lower QOL (31, 32). However, there are some shortcomings in the existing studies. On the one hand, they rarely pay attention to the relationships between impulsivity and QOL, and on the other hand, they did not determine whether there is a different relationships between the various domains of QOL (i.e., QOL is basically divided into four domains, namely physical, psychological, social, and environmental, see Method Section). In view of this, our research will focus on solving all these problems ignored by previous studies.

In addition to impulsivity, there are other factors that were consistently associated with the QOL of patients with SUDs, of which the most commonly studied were the characteristics related to drug use, including the frequency of drug use (33, 34), the severity of SUDs (35, 36), comorbidities with mental disorders (37, 38) and polysubstance use (39). In fact, in addition to the above-mentioned drug use characteristics, there are also the age of onset of SUDs, the number of relapse and comorbidities with other SUDs, which seem to have been overlooked in previous studies. Overall, the main purpose of this study was to identify the degree to which impulsivity and some previously less discussed drug use characteristics explain the variations in QOL, especially to clarify the relationships between these clinical variables and QOL in different domains. It is worth emphasizing that most previous studies have used the Addiction Severity Index (ASI) to assess the severity of SUDs (35, 36), which may produce some bias due to the self-reported data. In order to modify this, we obtained the severity of SUDs through the semi-structured interviews in this study, according to the diagnostic criteria of SUDs in DSM-5. In addition, the patients we recruited were recent users of methamphetamine (MA), as MA has increasingly become an illegal drug of abuse worldwide, especially in Asia (40). Currently, methamphetamine use disorder (MAUD) has become an global public health issue (41). Therefore, improving the QOL of MAUD patients is critical to prevent an outbreak.

## Method

### Participants and Procedures

A total of 379 MA users participated in this study, including of 324 men (85.5%) and 55 women (14.5%), aged from 20 to 54 years old (M =33.93 ± 7.08). They were recruited from two compulsory drug rehabilitation centers in Changsha, Hunan Province. From March to September 2018, two psychiatrists who validated the raters’ consistency conducted semi-structured interviews with MA users who entered drug rehabilitation centers. The inclusion criteria for this study were 1) the core drug used in the last three years was MA; 2) diagnosed as a stimulant use disorder (that is, meeting at least 2 criteria in DSM-5); 3) at least two weeks of detoxification at the time of enrollment. The exclusion criteria were 1) lifetime/current diagnosis of mental illness or personality disorder; 2) alcohol use disorder; 3) intellectual disability or cognitive impairment; 4) presence of any other serious diseases.

This study was approved by the Ethics Committee of The Second Xiangya Hospital of Central South University. The subjects understood the purpose and procedure of the study and signed an informed consent for. They could quit unconditionally and all information was confidential.

### Measures

#### Demographics and Drug Use Characteristics

To ensure data quality, we administrated a semi-structured interview to collect demographics and drug use characteristics of the patients as follows: 1) demographic data included gender, age, education, marital status (whether married), whether they had children, who they lived with (family member or non-family member), employment (full-time or part-time job, unemployment); 2) drug use characteristics included the age of onset of MAUD, the number of relapse (i.e., reused drugs after at least three months of absolute withdrawal), comorbidities with other SUDs [i.e., the patients in this study were classified as simple MAUD, having comorbidities with opioid use disorder (OUD) and comorbid ketamine use disorder (KUD), depending on their diagnosis] and the severity of MAUD (i.e., patients who met six or more criteria were diagnosed as severe MAUD, and those with less than 6 criteria were diagnosed as mild to moderate according to the DSM-5).

#### Impulsivity

We used the Chinese version of Barratt Impulsiveness Scale-11 (BIS-11) to assess three aspects of impulsivity (42), namely no-planning impulsivity (i.e., the tendency to live an irregular life), motor impulsivity (i.e., act without considering the consequences) and cognitive impulsivity (i.e., defects in thought or difficulty in solving problems). BIS-11 is a self-reported scale of 30 items and is rated on a 5-point Likert scale, ranging from 1 “never” to 5 “always” (43). For the purpose of the current study, the total score was calculated, and a higher score indicates a higher level of trait impulsivity, which was validated in patients with MAUD in our previous studies (44, 45). In this study, the Cronbach’s α of the total scale was 0.909.

#### Quality of Life

The brief WHO Quality of Life Assessment (WHOQOL-BREF) was used to assess patients**’** QOL (1). It has been validated in patients with SUDs (14, 46). WHOQOL-BREF is a self-reported scale composed of 26 items, in which item 3 to 26 constitutes the individual**’**s four domains of QOL, namely physical QOL (PHYSQOL, seven items), psychological QOL (PSYCHQOL, six items), social QOL (SOCIALQOL, three items) and environmental QOL (ENVIRQOL, eight items). These items are scored with a Likert-5 point scale and all items are positively scored except for item 3, 4, and 26. The sum of each subscale was calculated, and the higher score indicates the higher QOL in corresponding filed. In addition, item 1 and 2 independently assess a person**’**s overall QOL and physical condition. Since we aimed to examine how clinical variables affect different aspects of QOL, we did not analyze these two overall items. The Cronbach**’**s α of the four subscales ranged from 0.615 to 0.808 in this study.

### Statistical Analysis

Descriptive statistics were used to describe demographic and drug use characteristics. Pearson correlation and univariate regression analysis were used to initially identify the relationship between impulsivity, drug use characteristics and QOL. Multiple linear regression analysis was used to further examine the association of the aforementioned clinical variables. Considering the differences in the four aspects of QOL, we executed four regression models with PHYSQOL, PSYHQOL, SOCIALQOL, and ENVIRQOL as dependent variables, and those measures with *p <*0.1 in the previous univariate regression analysis together with gender and age as independent variables. All analysis was performed using SPSS software (version 23.0) with a bilateral significance level <0.05.

## Results

### Description of Demographics and Clinical Variables

Demographic data is shown in **Table 1**. The majority (85.5%) of MAUD patients were male. Their average age and years of education were 33.93 (7.08) and 9.96 (2.70) respectively. Regarding their marital status, 175 (46.2%) were married, while others (53.8%) were not. When asked who they usually lived with, 250 (66.0%) reported their family members. In addition, 243 (64.1%) had at least one child. In regard to their employment, 229 (60.4%) had a full-time job, while others were part-time or unemployed.

**Table 1:** Demographics of the patients (N = 379).

Variables				M (SD)	N (%)
Gender					
male					324 (85.5)
female					55 (14.5)
Age				33.93 (7.08)	
Education (years)				9.96 (2.70)	
Married					
yes					175 (46.2)
no					204 (53.8)
Live with family member					
	yes				250 (66.0)
	no				129 (34.0)
Have children					
	yes				243 (64.1)
	no				136 (35.9)
Employment					
	full-time job				229 (60.4)
	part-time job/unemployment				150 (39.6)

**Table 2** shows the drug use characteristics and scores of BIS-11 and WHOQOL-BREF. First, patients reported that their age of onset of MAUD was 30.25 (7.26), with an average of relapse for 2.47 (1.01) times since the onset of MAUD. Regarding comorbidities with other SUDs, 227 cases (59.9%) were diagnosed without comorbidities (that is, simple MAUD), while 46 cases (12.1%) were diagnosed as comorbidities with OUD, and the remaining 106 cases (28.0%) were diagnosed with comorbidities with KUD. In terms of the severity of MAUD, the majority (73.9%) met more than six diagnostic criteria of DSM-5 and they were therefore diagnosed as severe. Then, as we can see in **Table 2**, the total score of BIS-11 was between 36 and 126, with an average score of 80.85 (15.95). Meanwhile, the sum of four domains of QOL was 14.91 (SD = 2.13, PHYSQOL), 13.35 (SD = 2.12, PSYCHQOL), 14.38 (SD = 2.59, SOCIALQOL), and 13.54 (SD = 2.30, ENVIRQOL), respectively.

**Table 2:** Drug use characteristics and scores of BIS-11 and WHOQOL-BREF (N = 379).

Variables		M (SD)	N (%)
Age of onset of MAUD		30.25 (7.26)	
Number of relapses		2.47 (1.01)	
Comorbidity with other SUDs			
	without		227 (59.9)
	comorbidity with OUD		46 (12.1)
	comorbidity with KUD		106 (28.0)
Severity of MAUD			
	mild to moderate		100 (26.4)
	severe		279 (73.6)
Total score of BIS-11 (0.909)^a^		80.85 (15.95)	
WHOOQOL-BREF (0.886)^a^			
	PHYSQOL (0.703)^a^	14.91 (2.13)	
	PSYCHQOL (0.615)^a^	13.35 (2.12)	
	SOCIALQOL (0.647)^a^	14.38 (2.59)	
	ENVIRQOL (0.808)^a^	13.54 (2.30)	

MAUD, methamphetamine use disorder; SUDs, substance use disorders; OUD, opioid use disorder; KUD, ketamine use disorder; BIS-11, Barratt Impulsiveness Scale-11; WHOQOL-BREF, Brief WHO Quality of Life Assessment; PHYSQOL, physical quality of life; PSYCHQOL, psychological quality of life; SOCIALQOL, social quality of life; ENVIRQOL, environmental quality of life.

a Cronbach’s α.

### Correlations of Drug Use Characteristics, Impulsivity With QOL

We conducted correlation and univariate regression analysis to initially explore the relationship between multiple clinical variables and QOL. First, Pearson’s correlation results are shown in **Table 3**. We found that the total score of BIS-11 was negatively correlated with QOL in all four domains, with a correlation coefficient ranging from 0.308 (SOCIALQOL, p < 0.01) to 0.467 (PHYSQOL, p < 0.01). Except for PSYCHQOL, the number of relapses was negatively associated with QOL in other three domains, with correlation coefficients of 0.187 (PHYSQOL, *p* < 0.01), 0.175 (SOCIALQOL, *p* < 0.01), and 0.108 (ENVIRQOL, *p* < 0.05), respectively.

**Table 3:** Correlations and univariate regression of impulsivity, drug use characteristics and QOL.

	PHYSQOL	PSYCHQOL	SOCIALQOL	ENVIRQOL
**Correlations**^a^				
age of onset of MAUD	0.021	0.045	-0.028	0.039
number of relapses	-0.187^**^	-0.079	-0.175^**^	-0.108^*^
total score of BIS-11	-0.467^**^	-0.459^**^	-0.308^**^	-0.438^**^
**Univariate regression**^b^				
severe MAUD	-0.157^**^	-0.235^***^	-0.190^***^	-0.205^***^
comorbidity with OUD^c^	-0.227^***^	-0.129^*^	-0.134^*^	-0.175^**^
comorbidity with KUD^c^	-0.041	-0.073	-0.037	-0.074

^*^p < 0.05; ^**^p < 0.01; ^***^p < 0.001.

QOL, quality of life; PHYSQOL, physical quality of life; PSYCHQOL, psychological quality of life; SOCIALQOL, social quality of life; ENVIRQOL, environmental quality of life; MAUD, methamphetamine use disorder; BIS-11, Barratt Impulsiveness Scale-11; OUD, opioid use disorder; KUD, ketamine use disorder.

a Pearson correlation coefficient.

b Standardized Beta coefficient.

c The reference was simple MAUD.

**Table 3** also presented the results of univariate regression. We firstly found that severe MAUD was negatively associated with QOL in all four domains, which had the highest correlation with PSYCHQOL (r = -0.235, *p* < 0.001), and the lowest correlation with PHYSQOL (r = -0.190, *p* < 0.001). In addition, compared with the simple MAUD, patients comorbid with OUD had significantly lower QOL in all four domains, especially PHYSQOL (β = -0.227, *p* < 0.001).

### Multiple Linear Regression of Drug Use Characteristics, Impulsivity, and QOL

We performed four separate multiple regression models by using Stepwise and Enter methods. First, PHYSQOL was set as the dependent variable, and then gender, age, education, marital status, who lived with, employment, comorbidity with OUD or KUD, number of relapses, severity of MAUD, and impulsivity were set as independent variables. As shown in **Table 4**, impulsivity (β = -0.446, *p* < 0.001), number of relapse (β = -0.130, *p* = 0.004), full-time job (β = 0.093, *p* = 0.039) and comorbidity with OUD (β = -0.127, *p* = 0.008) explained 28.1% of the variation in PHYSQOL (F = 24.266, *p* < 0.001, adjusted R^2^ = 0.270).

**Table 4:** Multiple linear regression of impulsivity and drug use characteristics to QOL.

QOL domains	Predictors	β	T	*p*
PHYSQOL				
	impulsivity	-0.446	-9.953	<0.001
	number of relapses	-0.130	-2.863	0.004
	full-time job	0.093	2.071	0.093
	comorbidity with OUD	-0.127	-2.671	0.008
PSYCHQOL				
	impulsivity	-0.417	-8.822	<0.001
	married	0.177	3.842	<0.001
	severe MAUD	-0.140	-2.961	0.003
SOCIALQOL				
	impulsivity	-0.276	-5.478	<0.001
	number of relapses	-0.138	-2.795	0.005
	severe MAUD	-0.146	-2.884	0.004
	married	0.129	2.608	0.009
	age	-0.116	-2.190	0.029
ENVIRQOL				
	impulsivity	-0.387	-8.144	<0.001
	married	0.162	3.500	0.001
	full-time job	0.128	2.831	0.005
	severe MAUD	-0.106	-2.232	0.026

QOL, quality of life; PHYSQOL, physical quality of life; PSYCHQOL, psychological quality of life; SOCIALQOL, social quality of life; ENVIRQOL, environmental quality of life; MAUD, methamphetamine use disorder; OUD, opioid use disorder.

Second, we designated PSYCHQOL as dependent variable, and gender, age, marital status, whether or not have children, employment, comorbidity with OUD or KUD, severity of MA and impulsivity as independent variables. The results showed that 26.4% (F = 22.184, *p* < 0.001, adjusted R^2^ = 0.252) of the variation in PSYCHQOL were explained by impulsivity (β = -0.417, *p* < 0.001), married (β = 0.177, *p* < 0.001) and severe MAUD (β = -0.140, *p* = 0.003).

In the next analysis, SOCIALQOL was set as a dependent variable, and gender, age, marital status, having children, who lived with, number of relapses, comorbidity with OUD or KUD, severity of MAUD and impulsivity were included in the regression model. We found that impulsivity (β = -0.276, *p* < 0.001), number of relapse (β = -0.138, *p* = 0.005), severe MAUD (β = -0.146, *p* = 0.004), married (β = 0.129, *p* = 0.009) and age (β = -0.116, *p* = 0.029) accounted for 16.3% of the variation in SOCIALQOL (F = 10.344, *p* < 0.001, adjusted R^2^ = 0.148).

Finally, ENVIRQOL was set as a dependent variable, while gender, age, marital status, having children, who lived with, employment, number of relapses, comorbidity with or KUD, severity of MAUD, and impulsivity were independent variables. The results showed that 26.2% of variation in ENVIRQOL (F = 18.790, *p* < 0.001, adjusted R^2^ = 0.248) was explained by impulsivity (β = -0.387, *p* < 0.001), married (β = 0.162, *p* = 0.001), full-time job (β = 0.128, *p* = 0.005) and severe MAUD (β = -0.106, *p* = 0.026).

It is worth mentioning that we conducted the Bonferroni test, which tested the deviation caused by multiple comparisons, and the results were still significant.

## Discussion

To the best of our knowledge, this is the first study to explore the relationship between impulsivity, drug use characteristics and QOL in the MAUD population. The main findings were as follows. First, certain demographic and a range of drug use characteristics, more specifically, age, marital status, employment, number of relapses, comorbidity with OUD as well as the severity of MAUD were correlated with different QOL. Second, impulsivity was negatively associated with QOL in all four domains among patients with MAUD. Finally, impulsivity, drug use characteristics and some demographics collectively explained the varying degrees of variation in the four domains of QOL.

Regarding demographics, age, married, and having a full-time job can predict one or more QOL domains. Specifically, married positively predicted three domains (ENVIRQOL, SOCIALQOL, and PSYCHQOL), while having a full-time job positively predicted PHYSQOL and ENVIRQOL. These were partially consistent with previous studies in patients with SUDs (39, 47, 48) and other mental disorders (49), indicating that stable social support is more constructive for positive perception of ENVIRQOL. In addition, age was negatively associated with SOCIALQOL which was consistent with one study in patients with depression (50). One of the possible reasons is that SOCIALQOL is involved in the sexual well-being of patients, which is generally reported as unsatisfactory in the elderly (51, 52). In terms of drug use characteristics, number of relapses, severe MAUD, and comorbidity with OUD negatively predicted at least one domain of QOL. First, more relapses predicted poorer PHYSQOL and SOCIALQOL, which was consistent with the findings of some recurrent diseases such as acne (53), depression (54) and SUDs (55). Our findings suggested that relapse was an important risk factor for QOL of SUDs patients, especially in their physical and social domains. Therefore, when treating patients with a higher relapse frequency, special attention should be paid to the recovery of their physical and social functions. Second, compared with simple MAUD, patients comorbid with OUD had significantly lower PHYSQOL. This indirectly supports previous studies that opioid abusers had a particularly poor physical QOL (56–58), even lower than other SUDs (59). In fact, previous studies have also found that comorbidities with alcohol use disorders are a risk factor for QOL (60). Collectively, clinicians should pay special attention to patients with multiple SUDs because their potentially low quality of life may affect the outcomes. Finally, most QOL domains in patients with severe MAUD were worse than those patients with mild to moderate MAUD, confirming the results of previous studies that severity is a stable predictor of poor QOL no matter what assessments was used (35, 61).

In addition to the above results, there were more important findings supporting the previous conclusion that impulsivity was closely related to QOL in patients with impulse-related disorders (10, 22–24, 62), including patients with SUDs in this study. Specifically, impulsivity was significantly negatively correlated with different domains of QOL in patients with MAUD, and even after controlling for demographics and multiple drug use characteristics, impulsivity can still predict each different domain of QOL. This was consistent with our previous hypothesis that impulsivity was a powerful predictor of QOL in patients with SUDs (32, 63), while in the current study, MAUD patients (31, 64). In other words, patients with higher impulsivity have lower QOL. More interestingly, we found that although impulsivity was associated with QOL in all domains, the degree was different. The results of correlation and multiple linear regression showed that impulsivity was most closely related to PHYSQOL and least related to SOCIALQOL. This completely replicated the results of patients with bipolar disorder (22), suggesting that impulsivity was even a stable predictor of cross-domain QOL. Furthermore, impulsivity was the strongest predictor of PHYSQOL which refers to the patients’ physical pain, fatigue, sleep, mobility, daily life, dependence on medical treatment, and ability to work (65). Previous studies have found that patients with SUDs tend to report physical discomfort, such as pain (66, 67) and poor quality of sleep (68, 69), which may interfere with their treatment. Our findings may provide advice to clinicians that reducing impulsivity may improve their perception of PHYSQOL, thereby promoting treatment.

However, this study was not without limitations. First, all patients were recruited from compulsory drug rehabilitation centers, excluding those from voluntary drug rehabilitation centers. In fact, there were some differences in a variety of clinical variables between the two groups, which may lead different results. Second, there were fewer female patients in our sample, resulting an imbalanced sex ratio. Therefore, we did not perform a gender difference analysis when exploring these relationships. We will continue to supply female samples to achieve a balanced sex ratio and explore potential gender differences in our findings. In addition, this study was cross-sectional, and we cannot determine whether impulsivity is a longitudinal predictor of QOL in patients with MAUD. Therefore, additional research is needed to examine whether controlling impulsivity will improve the QOL of patients.

Despite these limitations, this study still has obvious advantages. We have identified that impulsivity can significantly predict all domains of QOL in patients with MAUD, even after controlling for other clinical variables. Our results, combined with previous studies, confirm that higher impulsivity is harmful to QOL of patients with impulsivity-related symptoms. In addition, we have also found that there are some differences between the predictors of different QOL domains. For example, comorbidity with OUD can only predict PHYSQOL. It is worth mentioning that, as the most populous country worldwide, our results are worthy of reference for other regions, especially those with large numbers of drug abusers. Overall, this study provides clinical guideline for treatments, that is, regulating impulsivity in patients with SUDs may help improve their QOL, and even maintain their drug rehabilitation.

## Data Availability Statement

The data analyzed in this study is subject to the following licenses/restrictions: The database can be obtained from the corresponding authors with any reasonable reasons. Requests to access these datasets should be directed to TL, liutieqiao123@csu.edu.cn.

## Ethics Statement

This study was approved by the Ethics Committee of The Second Xiangya Hospital of Central South University. The subjects understood the purpose and procedure of the study and signed an informed consent.

## Author Contributions

TL and WH designed the study. HS and XJZ supervised the whole process. QD designed the structure of the interviews and measures. YW, MQL, ZZ and LZ performed the semi-structure interviews and data collection. YZ and MYL participated in data collation. JZ computed statistical analyses and interpreted the results. YW managed the literature search and wrote the manuscript. XYZ designed the framework of the manuscript and revised it. All authors contributed to the article and approved the submitted version.

## Funding

The National Key R&D Program of China (2017YFC1310400), the National Natural Science Foundation of China (81371465 and 81671324) and the provincial Natural Science Foundation of Hunan (2020JJ4795) supported this study. The sponsors had no role in the study design, survey process, data analysis, and manuscript preparation.

## Conflict of Interest

The authors declare that the research was conducted in the absence of any commercial or financial relationships that could be construed as a potential conflict of interest.
